# Automated Assessment of Word- and Sentence-Level Speech Intelligibility in Developmental Motor Speech Disorders: A Cross-Linguistic Investigation

**DOI:** 10.3390/diagnostics15151892

**Published:** 2025-07-28

**Authors:** Micalle Carl, Michal Icht

**Affiliations:** Department of Communication Disorders, Ariel University, Ariel 40700, Israel

**Keywords:** speech intelligibility, automatic speech recognition (ASR), down syndrome, motor speech disorders, cross-linguistic assessment

## Abstract

**Background/Objectives**: Accurate assessment of speech intelligibility is necessary for individuals with motor speech disorders. Transcription or scaled rating methods by naïve listeners are the most reliable tasks for these purposes; however, they are often resource-intensive and time-consuming within clinical contexts. Automatic speech recognition (ASR) systems, which transcribe speech into text, have been increasingly utilized for assessing speech intelligibility. This study investigates the feasibility of using an open-source ASR system to assess speech intelligibility in Hebrew and English speakers with Down syndrome (DS). **Methods**: Recordings from 65 Hebrew- and English-speaking participants were included: 33 speakers with DS and 32 typically developing (TD) peers. Speech samples (words, sentences) were transcribed using Whisper (OpenAI) and by naïve listeners. The proportion of agreement between ASR transcriptions and those of naïve listeners was compared across speaker groups (TD, DS) and languages (Hebrew, English) for word-level data. Further comparisons for Hebrew speakers were conducted across speaker groups and stimuli (words, sentences). **Results**: The strength of the correlation between listener and ASR transcription scores varied across languages, and was higher for English (*r* = 0.98) than for Hebrew (*r* = 0.81) for speakers with DS. A higher proportion of listener–ASR agreement was demonstrated for TD speakers, as compared to those with DS (0.94 vs. 0.74, respectively), and for English, in comparison to Hebrew speakers (0.91 for English DS speakers vs. 0.74 for Hebrew DS speakers). Listener–ASR agreement for single words was consistently higher than for sentences among Hebrew speakers. Speakers’ intelligibility influenced word-level agreement among Hebrew- but not English-speaking participants with DS. **Conclusions**: ASR performance for English closely approximated that of naïve listeners, suggesting potential near-future clinical applicability within single-word intelligibility assessment. In contrast, a lower proportion of agreement between human listeners and ASR for Hebrew speech indicates that broader clinical implementation may require further training of ASR models in this language.

## 1. Introduction

The assessment of speech intelligibility among individuals with motor speech disorders is critical for determining the severity of the disorder and assessing the effectiveness of intervention programs [1]. However, reliable and unbiased measurement of speech intelligibility typically requires transcription-based or scaled ratings [2] by listeners who are naïve to the speaker [3] and to the motor speech disorder [4]. This process is often cumbersome and time-consuming, possibly impacting its use in clinical and research settings. The recent increase in the application of automated speech systems in the assessment of speech intelligibility among individuals with dysarthria (e.g., [5,6]) holds promise for creating an efficient method of intelligibility assessment for various clinical and research purposes. This potential is highlighted by the consistently increasing accuracy of such systems in speech recognition, even for individuals with motor speech disorders [7]. Nonetheless, most investigations published to date have been conducted in the English language, with limited data regarding the accuracy of such systems in languages other than English. The purpose of this study was to investigate the accuracy of an available automated speech-to-text system in comparison to naïve listeners for the purpose of intelligibility assessment among Hebrew- and English-speaking individuals with developmental motor speech disorders secondary to Down syndrome (DS).

### 1.1. Assessment of Speech Intelligibility

Speech intelligibility, in its narrow definition, refers to the degree to which the listener identifies the spoken symbol of a communicative partner [1,8], without the assistance of contextual cues [9]. From the perspective of the speaker’s overall functioning, speech intelligibility describes the speaker’s efficiency of communication with communicative partners [10]. Assessment of speech intelligibility skills, therefore, is an essential component of speech and language assessment procedures and allows for determination of the degree of impairment, as well as measuring change in functioning across times or conditions (e.g., following treatment) [1,10]. This is especially relevant for speakers with DS, a congenital, chromosomal disorder, for whom speech intelligibility is notably reduced across the lifespan [11,12].

The assessment of speech intelligibility can take many forms, including both transcription-based methods and measures of overall scaled ratings [8]. Scores obtained from these measures may be influenced by the type of stimulus elicited (e.g., words vs. sentences; [13]) and the listener’s experience (e.g., familiar vs. naïve listener; [14]). As related to the former, multi-word productions may be judged as more intelligible than single-word stimuli, attributed to the advantage of contextual clues within the sentence [13]. However, among speakers with motor speech disorders, the length and complexity of sentence production may limit this advantage, due to fatigue and overall load on the motor speech system [15,16]. Further interaction of the speech stimulus with speech intelligibility may occur among individuals with characteristics of motor planning impairment (apraxia of speech), as is noted among speakers with DS [17,18].

The use of transcription-based methods by naïve listeners is typically considered to be the “gold standard” of intelligibility assessment [19], although several have demonstrated correlation of such measures with other methods of assessment [20,21]. The benefits of transcription-based tasks further include the ability to analyze the results of these transcriptions, whether in open-set or closed-set formats (e.g., identification of the target word from a given choice of words; [1]), to determine phonetic elements or contrasts that are produced in error. Nonetheless, the inclusion of intelligibility transcriptions conducted by naïve listeners is not consistently used aside from research settings [19,22], as it requires time and resources that are not readily available to the practicing clinician. These limitations have prompted clinicians and researchers to explore alternative approaches for assessing speech intelligibility. Recent technological advancements have opened the door to utilizing automatic speech recognition (ASR) systems for this purpose.

### 1.2. Automatic Speech Technologies

The emergence of automatic speech recognition (ASR) technologies is transforming contemporary assessment and intervention practices in the field of communication disorders. The overall accuracy of speech recognition has increased over the past few years, even for individuals with speech disorders [23]. The use of automatic speech assessment or recognition technologies has been applied in various clinical applications, including feedback for children with speech–sound disorders [24,25], determination of disorder severity (e.g., [26]), and enhancing communication participation [27] (for a review, see [28]). However, the accuracy of such systems varies between studies, with noted difficulty for motor-based speech disorders (e.g., dysarthria; [28]). This lack of consistent accuracy has been attributed to the variability of speakers within and across disordered speaker populations, and the relatively limited speaker data that these systems are trained on [27,29]. Nonetheless, recent studies have demonstrated optimistic results, with improvement in ASR accuracy noted following the training of the automated systems on related stimuli [30,31].

An additional clinical application of automatic speech technologies is the assessment of speech intelligibility for speakers with motor speech disorders [5,6,32]. With many ASR systems trained primarily on typical speech, the question of whether the use of such technologies can simulate the performance of a naïve listener in judging (e.g., transcribing) disordered speech arises. A select number of studies have used available ASR platforms to determine their efficacy in judging speech intelligibility, in comparison to listeners. Gutz et al. [5] used the Google Cloud ASR API to judge speech intelligibility of adult speakers with Amyotrophic Lateral Sclerosis (ALS), and to determine the severity of the speech impairment. While the authors reported a strong correlation (*r* = 0.87) between listener and ASR word recognition rates (WRR), they noted that the latter did not differentiate between speaker severity levels. Similarly, Moya-Galé et al. [6] compared transcriptions by Google Cloud ASR API of sentences in noise to listeners for speakers with Parkinson’s disease (PD). Their results demonstrated a high probability (0.8) that the ASR platform would be as accurate (or better) than a human listener. Xue et al. [32] reported increased accuracy (up to *r* = 0.87) of the ASR-based intelligibility assessment when trained on some dysarthric speech, although results were compared to those from expert listeners. Finally, Tröger et al. [33] detailed moderate correlations (range of *r* = −32 to *r* = −46) between ASR-based intelligibility assessment scores and scores from clinical measures (e.g., dysarthria assessment), obtained from speakers with acquired dysarthria across several languages, as well as with those from typical (control) speakers, although they did not include comparisons with listeners.

The efficacy of utilizing ASR systems for speech intelligibility assessments is presumably related to the accuracy of such systems in transcribing typical speech. While languages trained on large-scale data sets (e.g., English) demonstrate high levels of transcription accuracy of typical speech [34], other languages demonstrate less accurate performance of the ASR system [35,36,37]. As such, utilizing open-source ASR platforms for assessment of speech intelligibility in disordered speech may differ in performance across languages. Furthermore, there is emerging evidence that the accuracy of ASR systems may be differentiated by the type of speech stimulus (e.g., read vs. conversational speech; [38]); this, too, may influence the accuracy of subsequent intelligibility assessments of disordered speech differing in stimulus type.

### 1.3. The Current Study

Given the potential of ASR systems in automating speech intelligibility assessments for disordered speech, the goal of the current study was to determine the efficacy of an open-source ASR platform, Whisper OpenAI (large v3) in measuring speech intelligibility for speakers with speech disorders secondary to DS. Although recent data has demonstrated promising results of automated intelligibility assessment, the variability between disorders and/or speaker groups [39] necessitates investigations for different speaker profiles. This is particularly highlighted for speakers with DS, in which speech profiles demonstrate characteristics of both dysarthria and apraxia of speech (disorder of motor planning and programming; [18,40]), in addition to the known within- and between-speaker variability associated with clinical groups [41]. Furthermore, cross-linguistic comparisons of ASR accuracy in relation to disordered speech are scarce. As such, the current study investigates the accuracy of word- and sentence-level transcriptions by Whisper OpenAI for both Hebrew and English speakers, with and without DS, in comparison to listener transcriptions. Research questions are listed as follows:

1. What is the level of accuracy of the ASR system for Hebrew- and English-speaking individuals with and without DS, in comparison to human listeners?
2. How does the proportion of agreement between ASR and listener intelligibility scores differ for each of the following factors: (a) language (Hebrew vs. English); (b) disorder (DS vs. typically developing (TD)); (c) stimulus type (word vs. sentence); (d) speech intelligibility?

## 2. Materials and Methods

The study was approved by the Ariel University ethics review board (#AU-HEA-MK-20240131). The current study used recordings of speakers with and without DS, as detailed in prior investigations [42,43]. Data obtained from English speakers were collected at the Graduate Center, CUNY, under IRB approval #2016-0665. As reported previously, informed consent for participation was obtained from all subjects or their parents/legal guardians involved in the current study. In addition, participants with DS provided verbal assent for partaking in the research procedures.

### 2.1. Participants

#### 2.1.1. Speakers

Recordings from Hebrew- and English-speaking young adults with DS, as well as from TD peers matched generally for age, were used in the current study. Hebrew speakers included 24 speakers with DS (13 males, 11 females), between the ages of 21 and 37 years, all of whom were fluent speakers of modern Hebrew and used speech as their primary means of communication. Hebrew-speaking TD participants included 24 native speakers of Modern Hebrew (12 males, 12 females) between the ages of 19 and 32 years. English speakers included eight speakers with DS (five males, three females) and six TD speakers (four males, two females) between 19 and 27 years of age. TD speakers self-confirmed typical hearing and a lack of speech or language disorders. Speakers with DS had no concomitant disorders (e.g., autism spectrum disorders) and reported no severe hearing impairment.

#### 2.1.2. Listeners

A total of 170 participants, between the ages of 18 and 60 years, served as naïve listeners for judgments of single-word and sentence stimuli. All listeners reported normal hearing, no concomitant speech or language impairment, as well as no extended experience with people with motor speech disorders. Listeners also reported their native language to be English or Hebrew, as per the language of the stimuli. For Hebrew stimuli judgments, a total of 144 listeners (127 female, 17 male) transcribed word- and sentence-level stimuli produced by speakers with and without DS. In order to avoid familiarity with the words and/or speech characteristics of speakers with DS, each listener transcribed only one word list. Listeners who transcribed single-word stimuli produced by TD speakers also listened to one set of sentences (15 different sentences) produced by TD speakers, as well as one set of sentences (approximately 15 sentences) produced by speakers with DS. For English stimuli judgments (single words), a total of 26 listeners (19 female, 7 male) transcribed word-level stimuli, of which some transcribed data from two speakers, whether speakers with DS or TD speakers (16 listeners total).

### 2.2. Tools and Materials

#### 2.2.1. Speech Samples

Hebrew. Speech stimuli included both word- and sentence-level productions, obtained from both speakers with DS and TD peers. Hebrew stimuli included a total of 46 single-word productions, of which 32 were from the Articulation and Naming test [44], a Hebrew articulation assessment. Sentence stimuli produced by speakers with DS were elicited during a picture description task [45], with an average length of utterance of 3.59 words. For Hebrew-speaking TD participants, sentence stimuli were taken from the Hebrew-adapted Frenchay Dysarthria Assessment-2 (FDA2) [46], in which sentences are three words in length. A total of five sentences per speaker were used for the current analyses. For four speakers with DS, only four sentences were elicited and used for the current analyses.

English. English stimuli included only word-level data; sentence data for English speakers across groups was not available for the current analyses. English word stimuli included 78 single-word productions from the Test of Children’s Speech intelligibility assessment (TOCS+) [47,48]. Word lists varied between English-speaking participants, as one was taken from the three available TOCS+ word lists.

Hebrew stimuli were recorded using a high-quality digital recorder (Philips DVT4010/DVT6110), placed approximately 8 cm from the speaker’s mouth. For Hebrew-speaking TD participants, a head-mounted microphone was used during the recording procedure. English stimuli were recorded via a head-mounted microphone (Audio-Technica AT8538 Power Module) directly onto the computer.

#### 2.2.2. Automatic Speech Recognition (ASR)

Whisper OpenAI was used for the automatic assessments of word and sentence stimuli. This open-source platform was chosen due to its availability, potential applicability in both research and clinical contexts, and overall transcription accuracy for both English and Hebrew [49]. Word and sentence stimuli were each uploaded and transcribed by the ASR system in a random order.

### 2.3. Procedures and Experimental Design

Recordings of both Hebrew- and English-speaking participants (speakers) were each part of a larger data collection and analysis protocol. Speakers were recorded in a quiet room, with stimuli presented on a screen. Speakers were instructed to name (single words) or describe (sentences for the DS group) the picture presented on the screen, and TD Hebrew speakers also read the target sentences. For English speakers, single-word productions were elicited with an auditory model in addition to the visual stimulus.

Listeners were recruited through the university as well as through word-of-mouth. The experimental listening task, which involved listening to audio recordings of words or sentences and transcribing them, was completed individually. Listeners were instructed to complete the task in a quiet environment and were given the option to listen to each recording up to two times. For Hebrew-speaking listeners, the transcription task took place using the web-based experimental platform Gorilla Experiment Builder (www.gorilla.sc) [50].

Each stimulus, whether word or sentence, was transcribed by a total of 3 listeners. Given the relatively short word list for Hebrew single-word stimuli, listeners transcribed only one word list, whether produced by speakers with DS or TD speakers, in order to prevent familiarity of the listener with the stimulus set. Listeners who transcribed word lists of TD Hebrew speakers also transcribed two distinct sets of sentences (Hebrew stimuli only), each consisting of approximately 15 different sentences: One set produced by TD Hebrew speakers and one set, containing different sentences, produced by Hebrew-speaking participants with DS.

Transcriptions obtained both from listeners and from the ASR platform were reviewed in order to calculate scores and agreement. Words that were phonemically identical to the target word were scored as correct, while omission or substitution of any part of a target word, including morphological elements, were scored as errors [6]. It should be noted that due to technical errors, sentence data was not recorded for two TD Hebrew speakers. Similarly, sentence data for four Hebrew-speakers with DS was not available as their productions included only single-word utterances.

### 2.4. Dependent Measures and Analysis

#### 2.4.1. Word Stimuli

A total of three listeners scored each speaker’s single-word productions in each language. The final single-word intelligibility score for each speaker was calculated as the average of the three scores. The correlation between the averaged listener intelligibility score and the ASR intelligibility score was calculated across languages and speaker groups. However, due to the lack of one-to-one mapping between the two, as reported in [5], the accuracy of the ASR transcriptions in relation to those of the listeners was determined by calculating the point-by-point proportion of agreement between the two. This was defined as the proportion of words that the ASR system transcribed in agreement with the listeners, whether correctly (in relation to the original stimulus) or incorrectly, out of the total number of words produced by that speaker. A word was scored as having “agreement” if at least two of the three listeners transcribed it as such (similar to the procedure in [24]). Although the term “word error rate” (WER), referring to a gold-standard transcription or reference, is commonly used in studies of ASR accuracy (e.g., [51,52]), the current study relied on the term “agreement” instead, to denote the similarity of scoring between human listeners and the ASR system for both correct and incorrect transcriptions in relation to the target word.

#### 2.4.2. Sentence Stimuli

Similar to single-word stimuli, a total of three listeners judged each speaker’s sentences. The scoring of sentences, as transcribed by listeners, as well as comparisons to ASR transcription of sentences, followed the procedures outlined in [6]: Transcriptions of all three listeners were scored by calculating the percentage of words correctly transcribed per sentence, for each listener. The percentage of correctly transcribed words per sentence was averaged across the three listeners, and then across the number of sentences, for the speaker’s final sentence intelligibility score (e.g., total score across sentences/number of sentences per speaker). Scores for ASR sentence transcriptions were also calculated as the percentage of correctly transcribed words per sentence, averaged across sentences, for a given speaker.

To determine the agreement between the listener and ASR transcriptions, the average proportion of “ASR successes” was calculated, defined as the proportion of instances that the ASR transcription score for any given sentence was equal to or greater than the listener-determined average score. This measure of agreement was then applied to the various statistical analyses to determine differences between speaker populations, stimulus type, and speech intelligibility. As noted above, sentences were recorded only for Hebrew speakers (TD and DS speaker groups).

### 2.5. Statistical Analysis

All statistical analyses were conducted in R (version 4.3.1) [53]. The relationship between listener and ASR transcription scores, for each of the word and sentence stimulus sets per language, was calculated via Pearson correlations. All further analyses were conducted on the listener–ASR proportion of agreement scores: To determine the effects of language (independent variable 1; Hebrew/English) and speaker group (independent variable 2; DS/TD) on the proportion of agreement between listener and ASR transcriptions (dependent variable), a two-way ANOVA was applied to the word-level data. To determine the effect of stimulus type on transcription agreement (listener–ASR) for Hebrew speakers only, a mixed-model ANOVA was calculated, with stimulus (word/sentence) as the within-group independent variable, and group (TD/DS) as the between-group independent variable. For each ANOVA model, post hoc analyses were applied in order to interpret the results using estimated marginal means (emmeans) [54] with pairwise comparisons and Tukey’s HSD correction for multiple comparisons. To further explore listener–ASR agreement scores as a function of intelligibility (as judged by listeners), a series of linear regressions (regression slopes and R^2^) were calculated on each subgroup (Language*Group for word data; Group*Stimulus for sentence data), with the Holm–Bonferroni method of correction for multiple comparisons [55]. This allowed a determination of whether the speech intelligibility score (i.e., the severity of the intelligibility impairment as indicated for speakers with DS) influenced the measure of listener–ASR agreement.

The normality of the listener–ASR agreement data was determined prior to the statistical analyses using measures of skewness and kurtosis, separately for word and sentence data. Both stimulus types demonstrated skewness values within ±2 and kurtosis values within ±7, suggesting that the data is normally distributed [56].

## 3. Results

Summary statistics of listener transcription scores, ASR transcription scores, and listener–ASR proportion of agreement scores, across variables (group, language, stimulus) are presented in Table 1.

### 3.1. Listener—ASR Correlations

**Figure 1:**
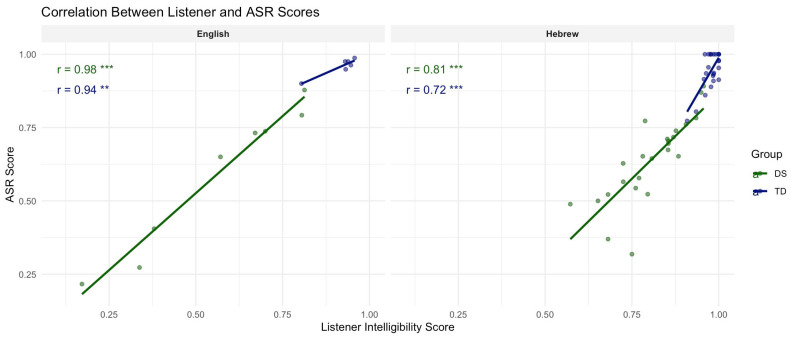
Word-level Pearson correlations between listener and ASR transcription intelligibility scores per speaker group (TD, DS) and language (English, Hebrew). *p*-values are denoted by asterisk (** < 0.01; *** < 0.001).

**Figure 2:**
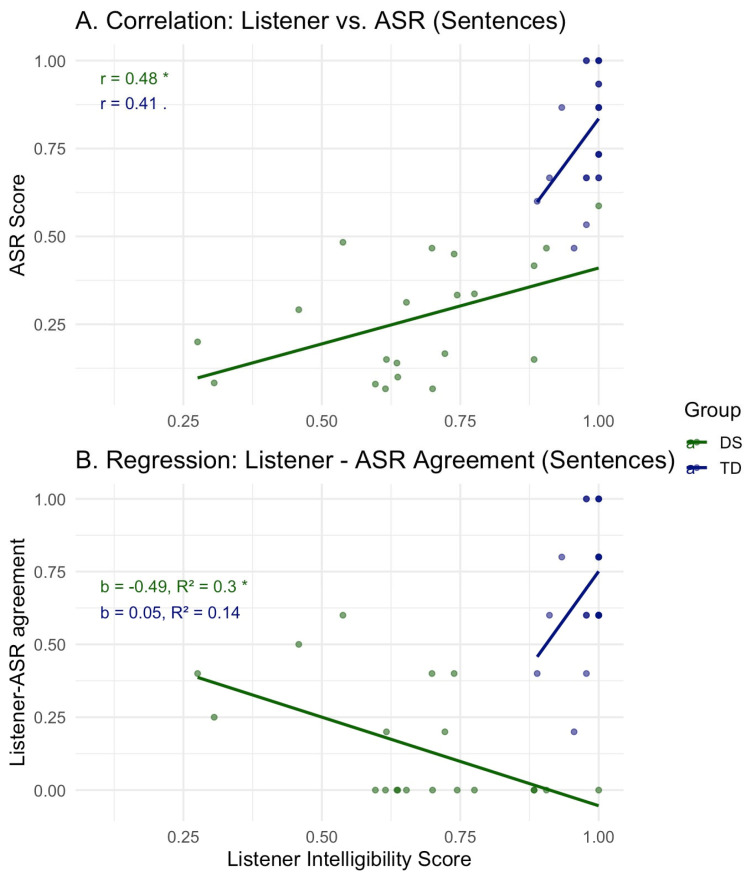
Sentence level analyses: (**A**) Pearson correlations between listener and ASR transcription intelligibility scores per speaker group; (**B**) Regression analyses of listener–ASR agreement and listener intelligibility scores per speaker group (TD, DS; for Hebrew speakers only). *p*-values are denoted by asterisk (* < 0.05).

Results of the Pearson product-moment correlations for word-level stimuli are reported in Figure 1. Strong correlations were demonstrated for both English-speaking groups (DS: *r* = 0.98, *p* < 0.001; TD: *r =* 0.94, *p* < 0.01), while correlations of comparatively reduced strength were reported for Hebrew-speaking groups (DS: *r* = 0.81, *p* < 0.001; TD: *r* = 0.72; *p* < 0.001). Pearson correlations for sentence-level stimuli produced by Hebrew speakers were of moderate strength for DS speakers (*r* = 0.48, *p* < 0.05), while an insignificant correlation was noted for TD speakers (*r* = 0.41, *p* = 0.06). For a visual depiction, see Figure 2A.

### 3.2. Listener—ASR Proportion of Agreement

Results of the two-way ANOVA revealed significant effects of both group, *F*(1, 58) = 43.35, *p* < 0.001, *η_p_*^2^ = 0.43, and language, *F*(1, 58) = 25.16, *p* < 0.001, *η_p_*^2^ = 0.3, on word-level agreement between listeners and ASR transcriptions, as well as an interaction between the two, *F*(1, 58) = 11.07, *p* < 0.01, *η_p_*^2^ = 0.16. Post hoc analysis using estimated marginal means, reported in Table 2, demonstrated significant contrasts between Hebrew and English speakers with DS, as well as between TD and DS Hebrew speakers. More specifically, agreement between listeners and ASR transcription was greater for English speakers with DS in comparison to Hebrew speakers with DS, as well as for TD Hebrew speakers in comparison to Hebrew speakers with DS. In contrast, no significant difference in listener–ASR agreement was noted between English speakers (TD vs. DS), as well as between TD speakers across the two languages. It should be noted that findings for English-speaking subgroups are based on a relatively small sample and should be interpreted with caution.

Results of the mixed-model ANOVA revealed significant effects of group, *F*(1, 40) = 120.65, *p* < 0.001, *η_p_*^2^ = 0.75, and stimulus, *F*(1, 40) = 147.68, *p* < 0.001, *η_p_*^2^ = 0.79, as well as an interaction between the two factors, *F*(1, 40) = 25.04, *p* < 0.001, *η_p_*^2^ = 0.39. Post hoc analyses using estimated marginal means, reported in Table 3, demonstrated significantly greater listener–ASR agreement for TD speakers in comparison to speakers with DS, for both word and sentence stimuli, as well as greater agreement for word stimuli in comparison to sentence stimuli, for both TD and DS speaker groups.

### 3.3. Regression Analysis: Proportion of Agreement and Listener Intelligibility

**Figure 3:**
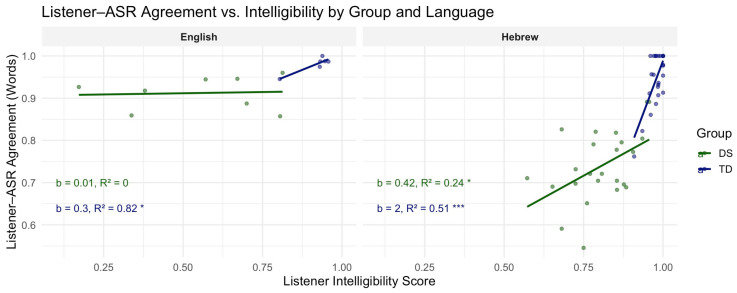
Regression analyses of word-level listener–ASR agreement and listener intelligibility scores per speaker group (TD, DS) and language (English, Hebrew). *p*-values are denoted by asterisk (* < 0.05, *** < 0.001).

A visualization of the linear regression models for word-level listener–ASR agreement scores relative to listener scores is presented in Figure 3. Significant positive relationships were noted between the two variables for three out of the four speaker groups: Hebrew speakers with DS, *b* (slope) = 0.415, *R*^2^ = 0.24, *p* < 0.05, Hebrew TD speakers, *b* = 2, *R*^2^ = 0.51, *p* < 0.001, and TD English speakers, *b* = 0.3, *R*^2^ = 0.82, *p* < 0.05. No significant relationship was noted for English speakers with DS, *b* = 0.01, *R*^2^ = 0.005, *p* = 0.87. For sentence-level data (Hebrew only), a significant, negative relationship was noted for DS speakers, *b* = −0.49, *R*^2^ = 0.3, *p* < 0.05, while no significant relationship was noted for TD speakers, *b* = 0.05, *R*^2^ = 0.14, *p* = 0.15 (see Figure 2B).

## 4. Discussion

The current study aimed to examine the accuracy of an open-source automatic speech recognition (ASR) system, Whisper OpenAI, in assessing the speech intelligibility of individuals with and without DS, in comparison to human naïve listeners. Specifically, the study sought to determine whether the proportion of agreement between listener and ASR transcriptions was influenced by speaker group (DS vs. TD), language (Hebrew vs. English), stimulus type (word vs. sentence, for Hebrew speakers only), and speech intelligibility score. Findings demonstrated stronger correlations between naïve listeners and ASR transcription scores for English speakers in comparison to Hebrew speakers, both with and without DS. The proportion of agreement between listener and ASR transcriptions was higher for English speakers than for Hebrew speakers, and higher for TD Hebrew speakers than for Hebrew speakers with DS. Agreement was also greater for word-level in comparison to sentence-level stimuli (measured for Hebrew speakers only). For the DS speaker group, the listener–ASR proportion of agreement was influenced by the severity of the intelligibility impairment for Hebrew- but not for English-speaking participants (word-level stimuli), as well as for sentences produced by Hebrew speakers with DS. While promising results were observed for English speakers, especially those with DS, the small sample size limits the strength of the conclusions and warrants cautious interpretation of clinical implications

The strong correlations between listener and ASR transcription scores for word-level stimuli in the current findings were generally expected, given similar findings for English [5,6], as well as in light of the increasing accuracy of such technologies over the past few years [7,38]. Nonetheless, the disadvantage of less prevalent languages (e.g., Hebrew) within the context of speech-to-text technologies is highlighted for sentence data in the current study, even for TD speakers. The current findings of reduced transcription accuracy of the ASR system in comparison to listeners, for sentence data specifically, indicate the importance of increasing data training sets for such technologies in order to improve recognition of stimuli varying in length and complexity. This finding may also be related to the type of sentence stimuli used for Hebrew TD speakers, in which words within the sentences were not predictable [46], leading to greater difficulty for ASR transcriptions in comparison to single-word data. In contrast, correlations between listener and ASR transcriptions of single-word stimuli were higher for both English TD (*r* = 0.94) and Hebrew TD (*r* = 0.72) speakers.

The lack of a one-to-one correlation between listener and ASR intelligibility scores, both in previous research (e.g., [5]) and within the current study, prompted a detailed analysis of agreement between the two measures. As expected, the proportion of agreement for word stimuli was greater for English than for Hebrew speakers overall, particularly among speakers with DS. However, two clinically relevant findings were demonstrated for English speakers: First, a lack of differentiation of the proportion of agreement was noted between TD and DS speakers; and second, impairment severity (denoted by speech intelligibility scores) among English speakers with DS did not impact this agreement. In other words, these findings imply that the accuracy of Whisper-OpenAI for the English language is nearly sufficiently high, such that it agrees with a listener across both typical and disordered speech and is not affected by the severity of the impairment. Although not investigated directly, this latter finding suggests that the ASR system may also provide an accurate index of intelligibility impairment severity, similar to those obtained from ratings of listeners [5]. In contrast, the findings for Hebrew speakers indicate that further accuracy for the ASR system in general is necessary before it can be used as a means of assessing speech intelligibility in a manner comparable to naïve listeners.

The results of stimulus comparisons (e.g., word vs. sentence stimuli) within Hebrew speakers, with and without DS, highlight the advantage of listeners, particularly for disordered speech. Listener–ASR agreement was significantly reduced for sentences in comparison to word-level stimuli, particularly for speakers with DS. Furthermore, the proportion of agreement between listener and ASR transcriptions was affected by the severity of the intelligibility impairment, although in the opposite direction as for word-level stimuli. Taken together, these findings indicate that stimuli with even slightly reduced accuracy, such as those found in connected speech [15,57], can pose a greater challenge for ASR systems in comparison to shorter, single-word stimuli. Furthermore, these results also underscore the human listener advantage of semantic inferencing and/or syntactic context in identifying and transcribing multi-word stimuli (e.g., sentence-level data) [13,15]. The context of mildly degraded sentence-level stimuli for speakers with DS, in which logical sequences of words were expected, may have facilitated the listener’s comprehension despite slight syntactic irregularities, but posed a challenge for the automated transcription. In contrast, sentences that were distinctly unintelligible garnered agreement, albeit for incorrect transcriptions, between listeners and the ASR system.

### 4.1. Clinical Implications

The findings of the current study offer important insights into the clinical application of ASR technologies in the field of speech-language pathology. Based on the limited sample of English-speaking individuals with DS, ASR systems such as Whisper OpenAI may show promise as potential tools for estimating speech intelligibility, potentially reducing the need for labor-intensive listener transcriptions in clinical and research settings. This may include, but is not limited to, determining the severity of speech intelligibility impairment, documenting change following interventions, and making comparisons with experienced or familiar listeners. However, caution is warranted, as broader validation across larger and more diverse clinical populations is necessary before automatic assessments fully replace listener evaluations.

For languages such as Hebrew, current ASR systems are not yet sufficiently accurate for clinical use within intelligibility assessment, particularly for speakers with motor speech disorders. Continued advancements in ASR model training in less prevalent languages such as Hebrew, even for typical speech alone, are essential to close this gap. These may include training models on larger datasets, in which there is a large sampling of the phonetic features, syntactic inflections, and phonotactic patterns that are unique to Hebrew.

### 4.2. Limitations and Future Directions

Several limitations of the current study should be acknowledged. First, the sample size for English-speaking participants, and particularly TD speakers, was relatively small (eight speakers with DS, six TD) due to the unavailability of the recording conditions in which the original stimulus set was collected. While promising results were observed for English speakers, especially those with DS, the small sample size limits the generalizability of the findings and warrants cautious interpretation of clinical implications. Second, sentence-level analyses were conducted only for Hebrew speakers, precluding cross-linguistic comparisons at the sentence level, and differed in the elicitation task between TD and DS speakers, possibly introducing confounding variables. Future work should investigate ASR accuracy and listener–ASR agreement levels for multi-word, sentence-level, and conversational-level stimuli for English speakers as well as within cross-linguistic investigations. Moreover, investigations into the role of context and semantic predictability on listener–ASR agreement could further refine the use of ASR in assessing connected speech. Finally, the current study did not compare different platforms of ASR, but rather focused on a single model. Comparisons of speech-to-text platforms, which may differ in their underlying processing strategies and architecture, are certainly warranted within and across languages.

To improve generalizability and clinical validity of ASR use within speech intelligibility assessments, future studies should aim to include substantially larger and more heterogeneous samples of participants across multiple languages. Based on power considerations and intergroup variability, a minimum of 25 to 30 participants per subgroup (e.g., DS, TD), per language is recommended. Furthermore, diversity should encompass not only linguistic background, but also disorder etiology (e.g., DS, cerebral palsy, apraxia of speech), age, and severity of impairment.

It seems plausible that at some point in the not-so-distant future, automatic speech recognition technologies will outperform the typical, “naïve” listener and may give overly accurate transcriptions of disordered speech, particularly for recognition models trained on dysarthric speech [32] or perhaps on the speaker’s own speech samples (e.g., [58]). In these instances, they will be applicable as speaker-assisted technologies, while their use within intelligibility assessment as a replacement for naïve listeners will have to take place on untrained sets only. The continued study of this advancing and evolving technology is critical to its successful and meaningful integration within clinical settings.

## Figures and Tables

**Table 1:** Summary statistics of listener intelligibility scores, ASR intelligibility scores, and the proportion of agreement between listener and ASR transcriptions, across language, speaker group (TD, DS), and stimulus type (word, sentence). Sentence data is available for Hebrew speakers only.

			Hebrew		English	
			DS	TD	DS	TD
		*n*	24	24	8	6
Listener	Word	mean (SD)	0.8 (0.1)	0.98 (0.02)	0.56 (0.24)	0.91 (0.06)
		range	0.57–0.96	0.91–1	0.17–0.81	0.8–0.96
	Sentences	mean (SD)	0.67 (0.18)	0.98 (0.03)	--	
		range	0.28–1	0.88–1	--	
ASR	Word	mean (SD)	0.64 (0.14)	0.94 (0.06)	0.59 (0.25)	0.96 (0.03)
		range	0.32–0.89	0.77–1	0.22–0.88	0.9–0.99
	Sentences	mean (SD)	0.27 (0.17)	0.79 (0.17)	--	
		range	0.06–0.59	0.47–1	--	
Listener–ASR Agreement	Word	mean (SD)	0.74 (0.08)	0.94 (0.06)	0.91 (0.04)	0.98 (0.02)
		range	0.55–0.89	0.76–1.00	0.86–0.96	0.94–1.00
	Sentences	mean (SD)	0.15 (0.2)	0.7 (0.22)	--	
		range	0.0–0.6	0.2–1.0	--	

**Table 2:** Estimated marginal means of language (Hebrew, English) and speaker group (TD, DS) effects on listener—ASR proportion of agreement for word stimuli.

Contrast	Estimate	SE	df	*t* Ratio	*p* Value
DS English—TD English	−0.07	0.04	58	−1.85	0.26
DS English—DS Hebrew	0.17	0.03	58	6.26	<0.0001
TD English—TD Hebrew	0.04	0.03	58	1.13	0.67
DS Hebrew—TD Hebrew	−0.21	0.02	58	−10.51	<0.0001

**Table 3:** Estimated marginal means of stimulus (word, sentence) and speaker group (TD, DS) effects on listener—ASR proportion of agreement (Hebrew speakers only).

Contrast	Estimate	SE	df	*t* Ratio	*p* Value
DS Sentence—TD Sentence	−0.55	0.07	40	−8.39	<0.0001
DS Sentence—DS Word	−0.59	0.05	40	−11.85	<0.0001
TD Sentence—TD Word	−0.25	0.05	40	−5.18	<0.0001
DS Word—TD Word	−0.21	0.02	40	−9.99	<0.0001

## Data Availability

The datasets presented in this article are not readily available because of confidentiality and privacy restrictions. Requests to access the datasets should be directed to the first author (M.C.).

## Abbreviations

The following abbreviations are used in this manuscript:

DS	Down syndrome
TD	Typically developing
ASR	Automatic speech recognition
